# Supplementary material for: Dual therapy with corticosteroid ablates the beneficial effect of DP2 antagonism in chronic experimental asthma
Source: Nat Commun. 2024 Nov 26;15:10253. doi: 10.1038/s41467-024-54670-8 (PMC11599388; doi:10.1038/s41467-024-54670-8)
Supplement: Supplementary file 1 — Supplementary Information [file 41467_2024_54670_MOESM1_ESM.pdf]

TITLE: Dual therapy with corticosteroid ablates the beneficial effect of DP2 antagonism in chronic experimental asthma.

Md Ashik Ullah[1], Sonja Rittchen[1,2], Jia Li[1,3], Bodie F. Curren[1,4], Patricia Namubiru[1,4], Tufael Ahmed[1], Daniel R. Howard[1,4], Muhammed Mahfuzur Rahman[1,4], Md Al Amin Sikder[1,4], Ridwan B. Rashid[1,4], Natasha Collinson[1], Mary Lor[1], Mark L. Smythe[5], Simon Phipps[1,4,6,7].

[1] Respiratory Immunology Laboratory, QIMR Berghofer Medical Research Institute, Herston, Queensland 4006, Australia;

[2] Otto Loewi Research Center for Vascular Biology, Immunology and Inflammation, Division of Pharmacology, Medical University of Graz, Graz 8010, Austria;

[3] Department of Laboratory Medicine, Shanghai General Hospital, Shanghai Jiao Tong University School of Medicine, Shanghai, 20080, China;

[4] School of Biomedical Sciences, University of Queensland, Queensland 4072, Australia;

[5] Institute for Molecular Bioscience, University of Queensland, Queensland 4072, Australia;

[6] Australian Infectious Diseases Research Centre, University of Queensland, Queensland 4072, Australia.

[7] School of Biomedical Sciences, Queensland University of Technology, Queensland 4000, Australia;

SUPPLEMENTARY FIGURES

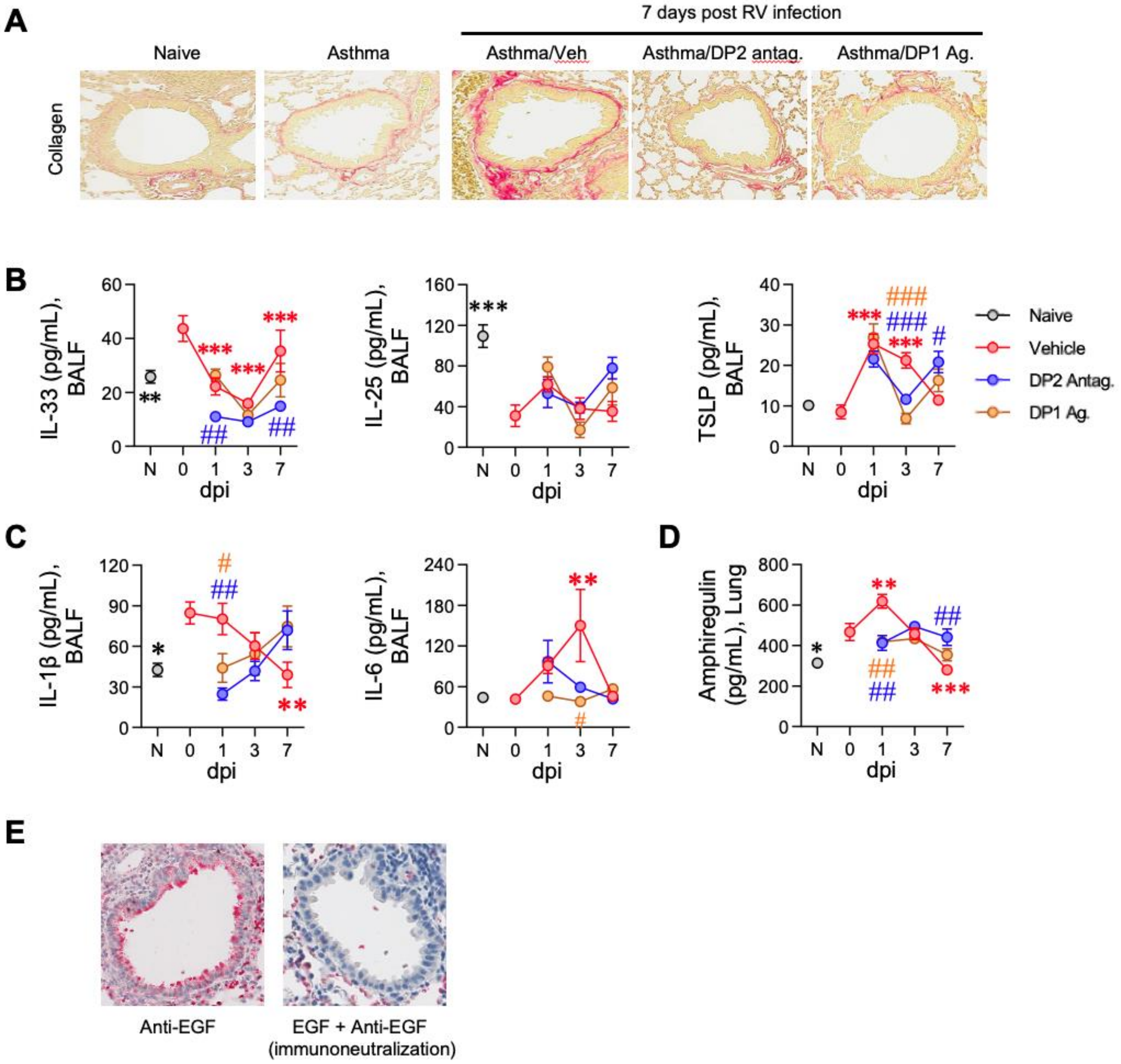

FIGURE S1. DP2 antagonism or DP1 agonism modulates the inflammatory response to RV-inoculation in mice with CEA.

Mice with CEA were inoculated with RV-1b and treated daily with a DP2 antagonist, a DP1 agonist, or vehicle starting from day 99. Mice were euthanized at 1, 3 and 7 dpi. (A) Representative lung histology of collagen expression (picrosirius red staining). (B-D) Cytokines were measured in the BALF or lung homogenates as indicated on the Y axis. (E) Specificity of EGF immunoreactivity was confirmed by preincubation of the antibody with exogenous EGF. Data are presented as mean ± SEM and are

representative of two independent experiments showing similar results (n = 4-11 mice per group). Statistical significance between different time points or different groups was determined using one-way ANOVA with Dunnett's multiple comparison test. * denotes $p<0.05$; ** denotes $p<0.01$ and *** denotes $p<0.001$ compared to Vehicle group. # denotes $p<0.05$, ## denotes $p<0.01$ and ### denotes $p<0.001$ compared to RV-infected group at corresponding time point. Source data are provided as a Source Data file.

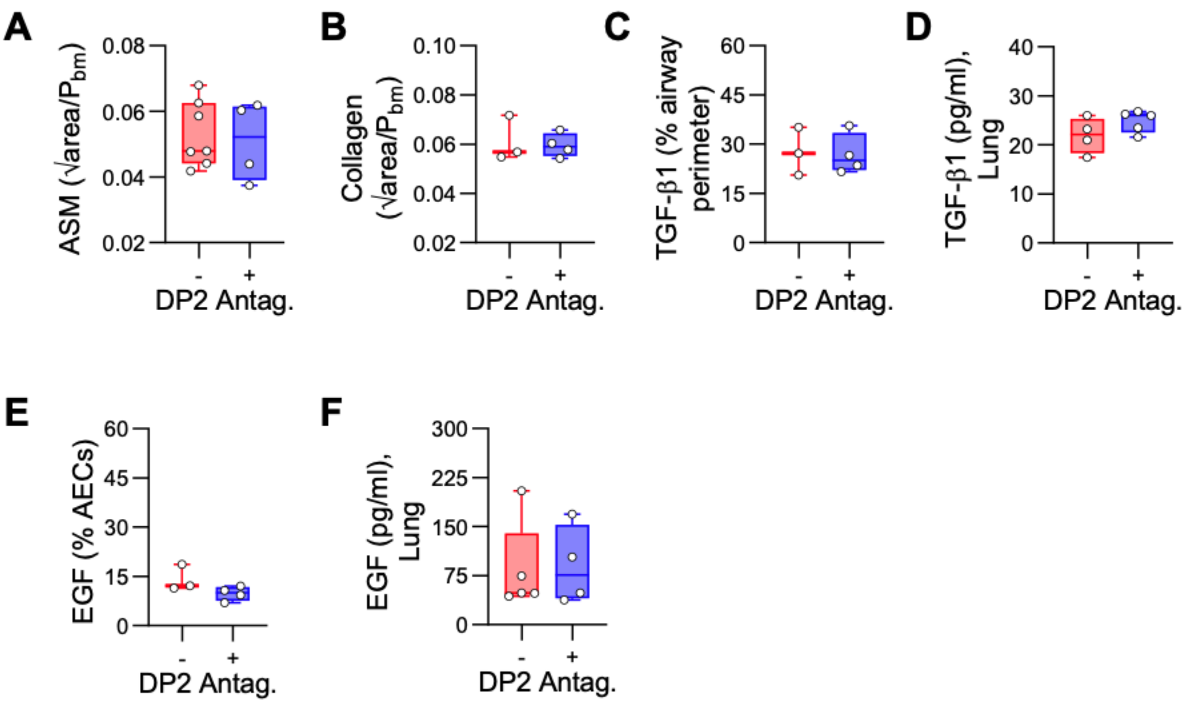

FIGURE S2. DP2 antagonism does not ameliorate airway remodelling in mice with chronic asthma.

Mice with chronic asthma were treated with DP2 antagonist, OC000459 daily from day 99 and were euthanized on day 108. (A) ASM area. (B) Collagen area. (C) Quantification of TGF-β1 immunoreactivity around airways. (D) TGF-β1 levels in lung homogenates. (E) Quantification of EGF immunoreactivity in AECs. (F) EGF levels in lung homogenates. Data are presented as box-and-whisker plots showing individual data points with the boxes representing quartiles and whiskers indicating the range and are representative of two independent experiments showing similar results (n = 3-7 mice per group). Group differences were analyzed using the Mann-Whitney U test. Source data are provided as a Source Data file.

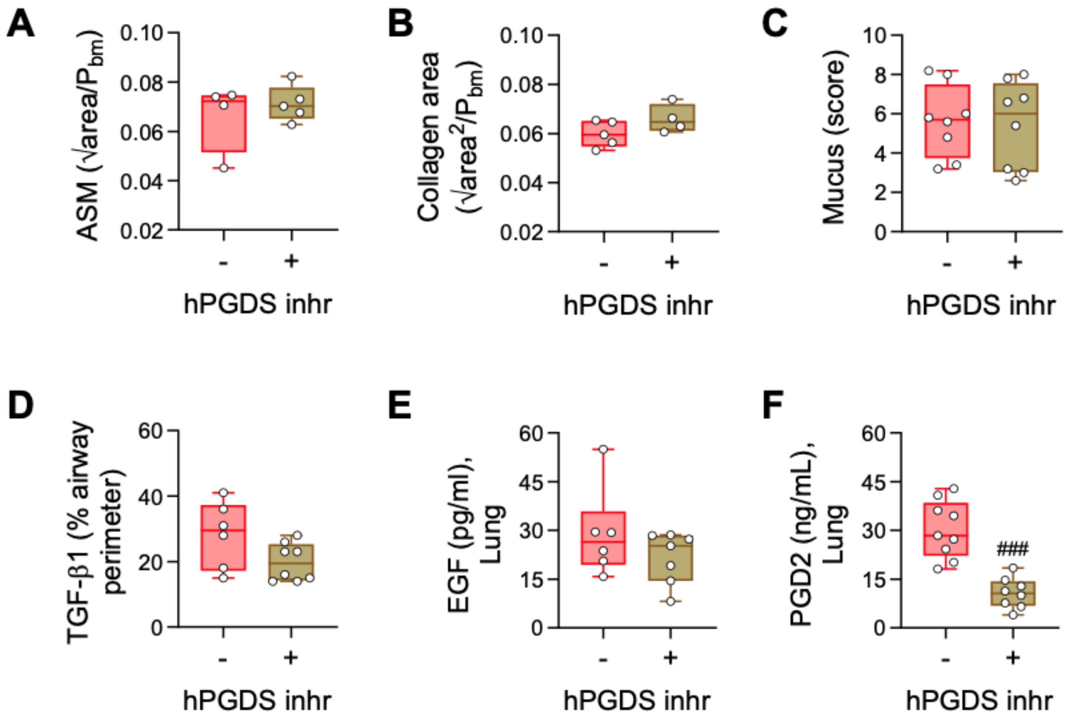

FIGURE S3. Inhibition of h-PGDS does not resolve airway remodelling during RV-induced exacerbation of chronic asthma.

Mice with chronic asthma were inoculated with RV-1b on day 101. Separate group of RV-1b infected mice were treated with h-PGDS inhibitor (PK007) daily from day 99 onwards and euthanized 3 days post infection. (A) ASM area. (B) Collagen area. (C) Muc5ac score. (D) Quantification of TGF-β1 immunoreactivity around airways. (E) Lung EGF levels in homogenates. (F) Lung PGD2 levels. Data are presented as box-and-whisker plots showing individual data points with the boxes representing quartiles and whiskers indicating the range and are representative of two independent experiments showing similar results (n = 4-9 mice per group). Group differences were analyzed using the Mann-Whitney U test. ### denotes p<0.001. Source data are provided as a Source Data file.

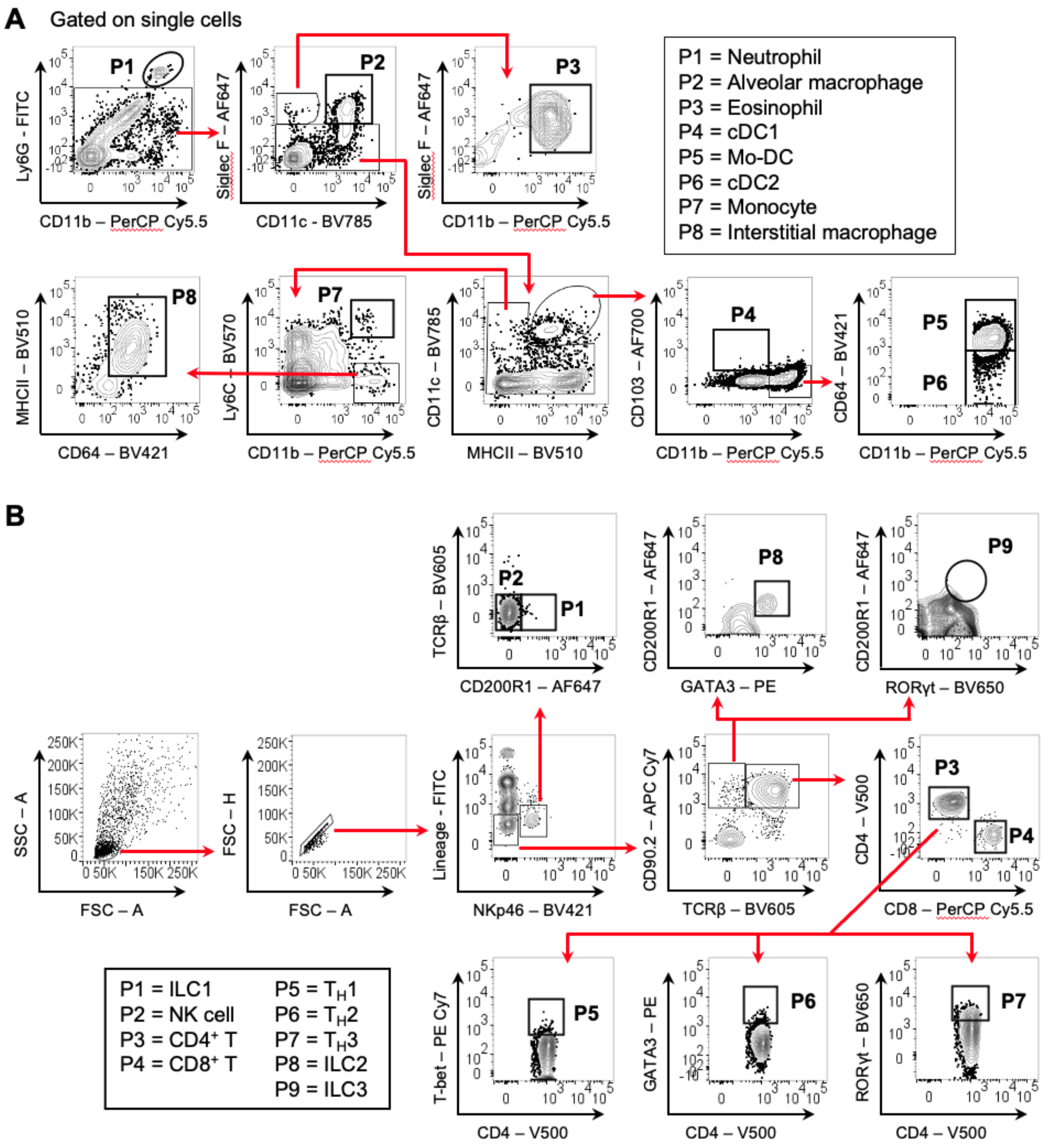

FIGURE S4. Gating strategies of immune cells in the lung.

Representative flow cytometry plots depicting the gating strategies for lung immune cells.

FIGURE S5.

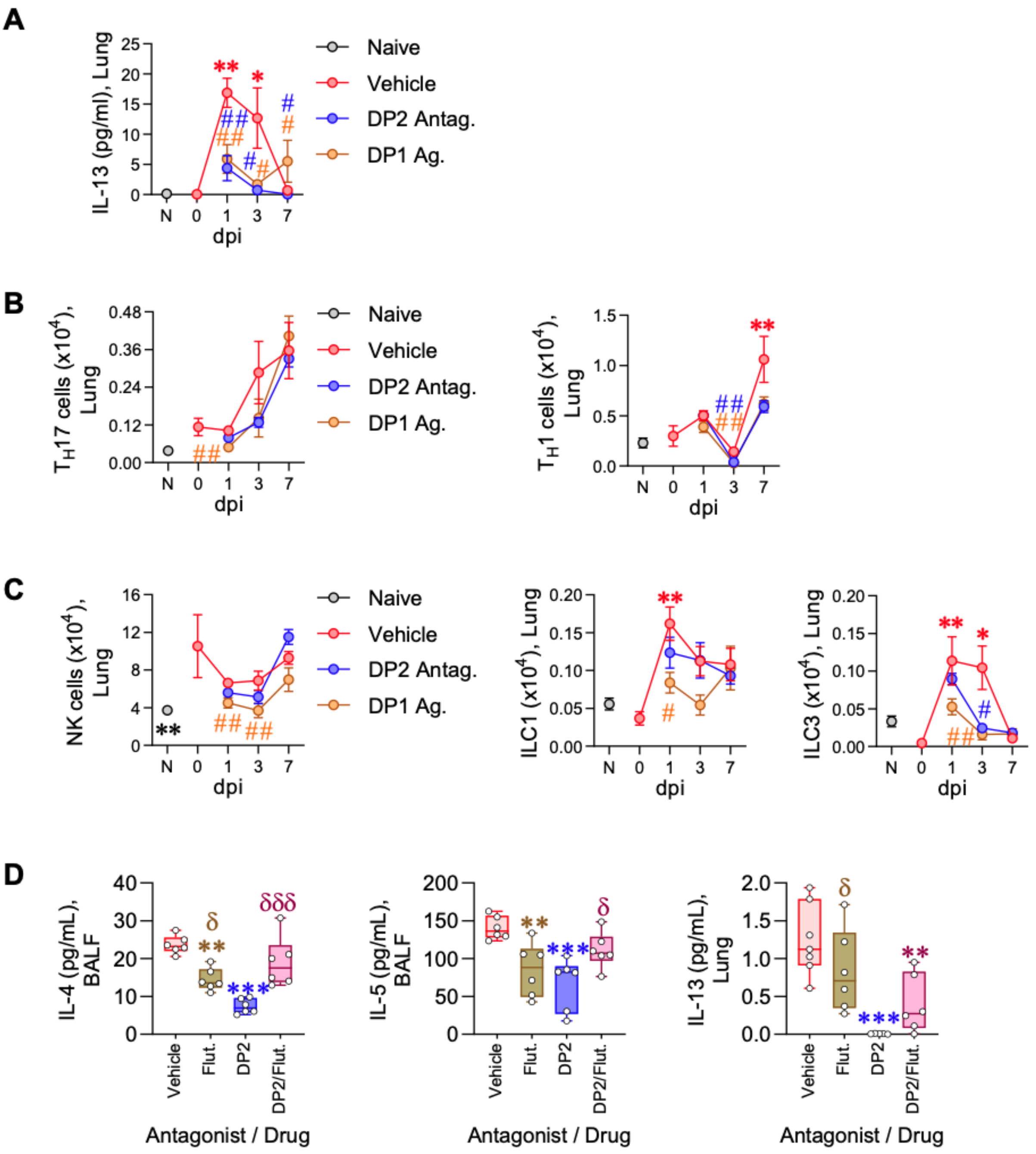

FIGURE S5. Effect of drug treatment on RV-induced inflammation in mice with CEA.

Mice with chronic asthma were inoculated with RV-1b and treated with DP2 antagonist or DP1 agonist or vehicle daily starting from day 99. Mice were euthanized at 1, 3 and 7 dpi. (A) IL-13 expression in the lungs. (B) Number of T_H17 (RORγt$^+$CD4$^+$ T) and T_H1 (T-bet$^+$CD4$^+$ T) cells in the lungs. (C) Number of NK cells

(CD3ε^{-}CD19^{-}NK1.1^{+}NKp46^{+}CD200R1^{-}), ILC1s (CD3ε^{-}CD19^{-}CD45R^{-}CD11c^{-}Gr-1^{-} NK1.1^{+}NKp46^{+}CD90.2^{+}CD200R1^{+}T-bet^{+}) and ILC3s (CD3ε^{-}CD19^{-}CD45R^{-}CD11c^{-} Gr-1^{-}CD11b^{-} CD90.2^{+}CD200R1^{+}RORγt^{+}) in the lungs. Data are presented as mean ± SEM and are representative of two independent experiments showing similar results (n = 4-7 mice per group). * denotes p<0.05 and ** denotes p<0.01 compared to Vehicle group. # denotes p<0.05 and ## denotes p<0.01 compared to RV-infected group at corresponding time point. (D) RV-1b infected mice were treated with vehicle or DP2 antagonist or fluticasone or both DP2 antagonist and fluticasone daily from day 99 and euthanized at 7 dpi. Concentrations of IL-4 and IL-5 in the BALF and IL-13 in the lungs. Data are presented as box-and-whisker plots showing individual data points with the boxes representing quartiles and whiskers indicating the range and are representative of two independent experiments showing similar results (n = 4-7 mice per group). Statistical significance between different time points or different groups was determined using one-way ANOVA with Dunnett's multiple comparison test. * denotes p<0.05; ** denotes p<0.01 and *** denotes p<0.001 compared to vehicle group. δ denotes p<0.05 and δδδ denotes p<0.001 compared to DP2 antagonist (OC000459) treated group. Source data are provided as a Source Data file.

FIGURE S6.

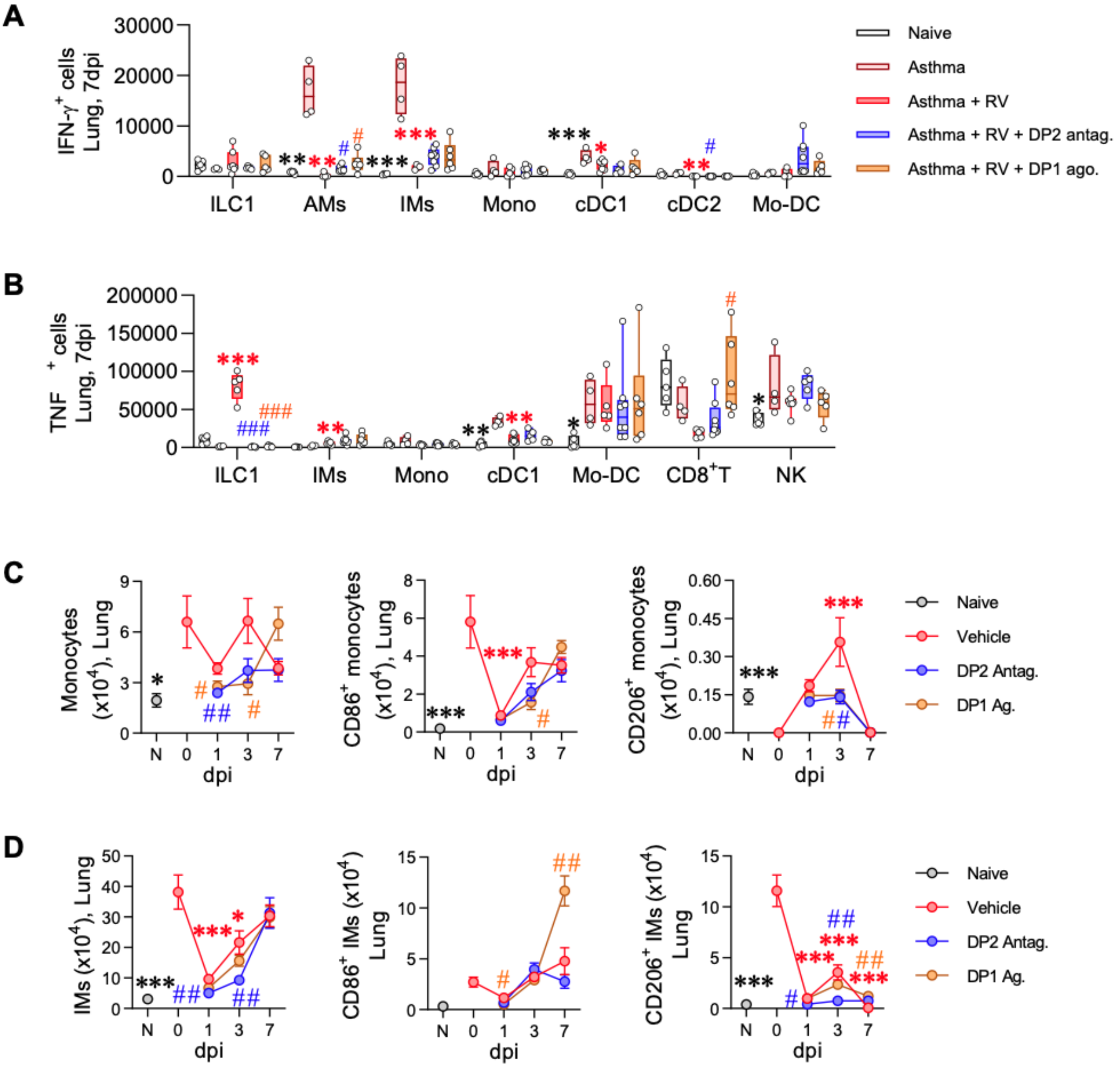

FIGURE S6.

Mice with chronic asthma were inoculated with RV-1b and treated with a DP2 antagonist or a DP1 agonist daily starting from day 99. Mice were euthanized at 1, 3 and 7 dpi. (A) Number of IFN-γ expressing ILC1, AMs, interstitial macrophages (IMs), monocytes (Mono), conventional type-1 dendritic cells (cDC1), conventional type-2 dendritic cells (cDC2) and monocyte-derived dendritic cells (Mo-DC) in the lungs at 7 dpi. (B) Number of TNF expressing ILC1, IMs, Mono, cDC1, Mo-DC, $CD8^{+}$T and NK cells in the lungs at 7 dpi. (C) Number of total monocytes ($CD11b^{+}Ly6C^{+}SiglecF^{-}$) and

CD86-expressing and CD206-expressing monocytes in the lungs. (D) Number of total interstitial macrophages (IM; SiglecF^{-}Ly6C^{-}CD11b^{+}MHCII^{+}CD64^{+}) and CD86-expressing and CD206-expressing IMs in the lungs. Data are presented as mean ± SEM or box-and-whisker plots showing individual data points with the boxes representing quartiles and whiskers indicating the range and are pooled data from two independent experiments showing similar results (n = 4-8 mice per group). Statistical significance between different time points or different groups was determined using one-way ANOVA with Dunnett's multiple comparison test. * denotes p<0.05; ** denotes p<0.01 and *** denotes p<0.001 compared to asthma or vehicle group at 0 dpi. # denotes p<0.05, ## denotes p<0.01 and ### denotes p<0.001 compared to RV-infected group at corresponding time point. Source data are provided as a Source Data file.

FIGURE S7.

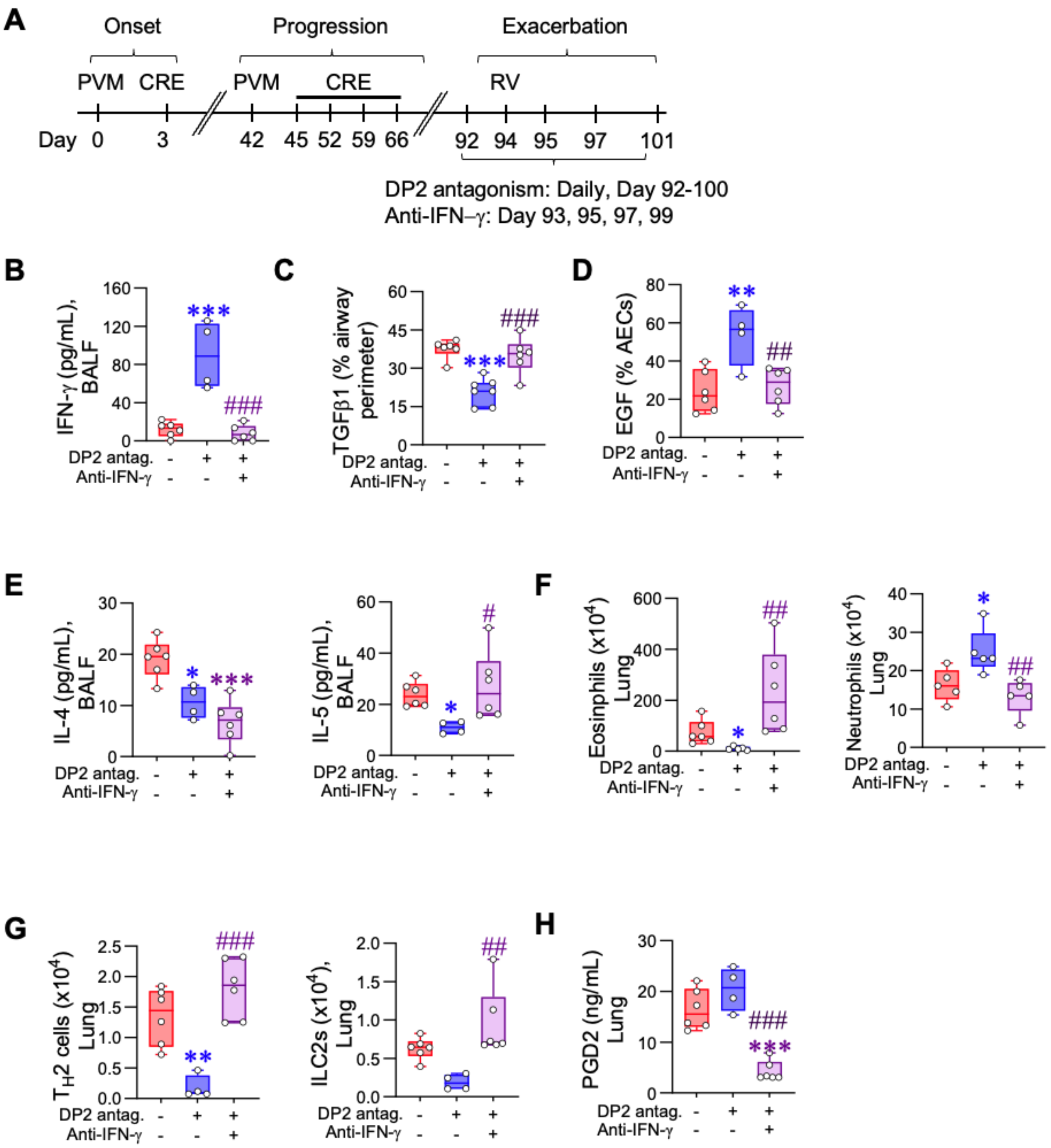

Figure S7.

(A) Study design. RV-1b infected mice were given vehicle or DP2 antagonist and treated with anti-IFN-γ. Mice were euthanized at 7 dpi. (B) IFN-γ expression in the BALF. (C) Quantification of TGF-β1 immunoreactivity around the airways. (D) Quantification of EGF immunoreactivity in AECs. (E) Concentrations of IL-4 and IL-5 in the BALF. (F) Number of eosinophils and neutrophils in the lungs. (G) Number of

T_H2 cells and ILC2s in the lungs. (H) Lung PGD2 levels. Data are presented as box-and-whisker plots showing individual data points with the boxes representing quartiles and whiskers indicating the range and are representative of two independent experiments showing similar results (n = 4-6 mice per group). Statistical significance between different groups was determined using one-way ANOVA with Dunnett's multiple comparison test. * denotes p<0.05; ** denotes p<0.01 and *** denotes p<0.001 compared to vehicle group. # denotes p<0.05, ## denotes p<0.01 and ### denotes p<0.001 compared to OC000459-treated group. Source data are provided as a Source Data file.

SUPPLEMENTARY TABLE

Table S1. Information of antibodies, kits and reagents used in this study

Name	Supplier
Anti-mouse antibodies	
B220-FITC (RA3-6B2)	Biolegend (CA, USA)
CD103-AF700 (2E7)	Biolegend (CA, USA)
CD11b-PerCp-Cy5.5, BV605 (M1/70)	BD Biosciences (CA, USA)
CD11c-BV785 (N418)	Biolegend (CA, USA)
CD19-FITC (1D3/CD19)	Biolegend (CA, USA)
CD200R1-AF647 (OX-110)	BD Biosciences (CA, USA)
CD206-PECy7 (C068C2)	Biolegend (CA, USA)
CD3ε-FITC (17A2)	Biolegend (CA, USA)
CD4-FITC (RM4-5)	Biolegend (CA, USA)
CD4-V500 (RM4-5)	BD Biosciences (CA, USA)
CD64-BV421 (X54-5/7.1)	Biolegend (CA, USA)
CD8-PerCp-Cy5.5 (53-6.7)	Biolegend (CA, USA)
CD80-APC/Fire750 (16-10A1)	Biolegend (CA, USA)
CD86-PE (GL-1)	Biolegend (CA, USA)
CD90.2-APC Cy7 (53-2.1)	BD Biosciences (CA, USA)
EGF	R&D systems (MN, USA)
F4/80-FITC, APC, APC Cy7 (BM8)	Biolegend (CA, USA)
FoxP3-AF647 (MF23)	BD Biosciences (CA, USA)
GATA3-PE (TWAJ)	eBiosciences (CA, USA)
Gr-1-FITC (RB6-8C5)	Biolegend (CA, USA)

IFN-γ-PE Cy7 (XMG1.2)	Biolegend (CA, USA)
Ly6C-BV570 (HK1.4)	Biolegend (CA, USA)
Ly6G-FITC (1A8)	BD Biosciences (CA, USA)
MHCII-BV510 (M5/114.15.2)	Biolegend (CA, USA)
Muc5ac (45M1)	Invitrogen (CA, USA)
NKp46-BV421 (29A1.4)	Biolegend (CA, USA)
RORγt-BV650 (Q31-378)	BD Biosciences (CA, USA)
Siglec F-PE (E50-2440)	BD Biosciences (CA, USA)
T-bet-PE Cy7 (4B10)	Biolegend (CA, USA)
TCR-β-BV605 (H57-597)	Biolegend (CA, USA)
TER119-FITC (TER-119)	Biolegend (CA, USA)
TGF-β1 (polyclonal)	Abcam (USA)
TNF-FITC (MP6-XT22)	BD Biosciences (CA, USA)
α-Smooth muscle actin (1A4)	Sigma-Aldrich (Mo, USA)
Kits	
FoxP3/TF fixation/permeabilization kit	eBiosciences (CA, USA)
Mouse Amphiregulin ELISA Kit	R&D systems (MN, USA)
Mouse EGF ELISA Kit	R&D systems (MN, USA)
Mouse IFN-γ ELISA Kit	Biolegend (CA, USA)
Mouse IL-13 enhanced sensitivity flex set	BD Biosciences (CA, USA)
Mouse IL-1β ELISA Kit	Biolegend (CA, USA)
Mouse IL-17A ELISA Kit	Biolegend (CA, USA)
Mouse IL-4 ELISA Kit	BD Biosciences (CA, USA)
Mouse IL-5 ELISA Kit	BD Biosciences (CA, USA)

Mouse IL-6 ELISA Kit	Biolegend (CA, USA)
Mouse IL-25 ELISA Kit	Biolegend (CA, USA)
Mouse IL-33 ELISA Kit	R&D systems (MN, USA)
Mouse TGF-β1 ELISA kit	R&D systems (MN, USA)
Mouse TSLP ELISA Kit	Biolegend (CA, USA)
Mouse TNF enhanced sensitivity flex set	BD Biosciences (CA, USA)
PGD2-MOX Express ELISA kit	Cayman Chemicals (Michigan, USA)
Zombie Aqua™ Fixable Viability Kit	Biolegend (CA, USA)
Reagents	
Brefeldin A	Biolegend (CA, USA)
BW245c	Cayman Chemical (MI, USA)
Cockroach extract	Greer Laboratories (NC, USA)
Hyclone fetal calf serum	Cytiva (USA)
Fluticasone	Sigma-Aldrich (MO, USA)
Glycerol gel	Sigma-Aldrich (MO, USA)
Hematoxylin, Dako	Agilent Technologies (Australia)
IGEPAL® CA-630	Sigma-Aldrich (MO, USA)
Ionomycin	Sigma-Aldrich (MO, USA)
MK-0524	Cayman Chemical (MI, USA)
OC000459	Cayman Chemical (MI, USA)
Phorbol 12-myristate 13-acetate (PMA)	Sigma-Aldrich (MO, USA)
Sirius red	Sigma-Aldrich (MO, USA)
Sodium chloride	Sigma-Aldrich (MO, USA)

Sodium deoxycholate	Sigma-Aldrich (MO, USA)
Sodium dodecyl sulfate	Sigma-Aldrich (MO, USA)
Soluble IL-13Rα2	Pfizer, NY, USA
Tris-base	Sigma-Aldrich (MO, USA)
