# Supplementary material for: Dual therapy with corticosteroid ablates the beneficial effect of DP2 antagonism in chronic experimental asthma
Source: Nat Commun. 2024 Nov 26;15:10253. doi: 10.1038/s41467-024-54670-8 (PMC11599388; doi:10.1038/s41467-024-54670-8)
Supplement: Supplementary file 2 — Reporting Summary [file 41467_2024_54670_MOESM2_ESM.pdf]

nature portfolio

Corresponding author(s): Simon Phipps

Last updated by author(s): Dec 12, 2023

Reporting Summary

Nature Portfolio wishes to improve the reproducibility of the work that we publish. This form provides structure for consistency and transparency in reporting. For further information on Nature Portfolio policies, see our Editorial Policies and the Editorial Policy Checklist.

Statistics

For all statistical analyses, confirm that the following items are present in the figure legend, table legend, main text, or Methods section.

n/a	Confirmed	
☐	☒	The exact sample size (n) for each experimental group/condition, given as a discrete number and unit of measurement
☐	☒	A statement on whether measurements were taken from distinct samples or whether the same sample was measured repeatedly
☐	☒	The statistical test(s) used AND whether they are one- or two-sided<br>*Only common tests should be described solely by name; describe more complex techniques in the Methods section.*
☐	☒	A description of all covariates tested
☐	☒	A description of any assumptions or corrections, such as tests of normality and adjustment for multiple comparisons
☐	☒	A full description of the statistical parameters including central tendency (e.g. means) or other basic estimates (e.g. regression coefficient) AND variation (e.g. standard deviation) or associated estimates of uncertainty (e.g. confidence intervals)
☐	☒	For null hypothesis testing, the test statistic (e.g. F, t, r) with confidence intervals, effect sizes, degrees of freedom and P value noted<br>*Give P values as exact values whenever suitable.*
☒	☐	For Bayesian analysis, information on the choice of priors and Markov chain Monte Carlo settings
☒	☐	For hierarchical and complex designs, identification of the appropriate level for tests and full reporting of outcomes
☒	☐	Estimates of effect sizes (e.g. Cohen's d, Pearson's r), indicating how they were calculated

Our web collection on statistics for biologists contains articles on many of the points above.

Software and code

Policy information about availability of computer code

Data collection	FlowJo software (Version 10.6; TeeStar, USA), Aperio AT Turbo (Leica Biosystems, Wetzlar, Germany), Aperio ImageScope software (Version 12.3.2.8013; Leica Biosystems, Wetzlar, Germany), GraphPad Prism (version 8, USA).
Data analysis	Images were scanned using the Aperio AT Turbo (Leica Biosystems, Wetzlar, Germany) and analyzed using the Aperio ImageScope software (Leica Biosystems, Wetzlar, Germany).<br>Flow cytometric analysis was performed using FlowJo software (Version 10.6; TeeStar, USA).<br>All graphs and statistical analysis were created and analysed via GraphPad Prism (version 8, USA).

For manuscripts utilizing custom algorithms or software that are central to the research but not yet described in published literature, software must be made available to editors and reviewers. We strongly encourage code deposition in a community repository (e.g. GitHub). See the Nature Portfolio guidelines for submitting code & software for further information.

Data

Policy information about availability of data

All manuscripts must include a data availability statement. This statement should provide the following information, where applicable:

- Accession codes, unique identifiers, or web links for publicly available datasets
- A description of any restrictions on data availability
- For clinical datasets or third party data, please ensure that the statement adheres to our policy

All the data required to support the findings in this report are included in the article. Extended data is provided in the Supplementary figures files and an expanded methods information is outlined in the supplementary methods file.

Research involving human participants, their data, or biological material

Policy information about studies with human participants or human data. See also policy information about sex, gender (identity/presentation), and sexual orientation and race, ethnicity and racism.

Reporting on sex and gender	n/a
Reporting on race, ethnicity, or other socially relevant groupings	n/a
Population characteristics	N/A
Recruitment	N/A
Ethics oversight	N/A

Note that full information on the approval of the study protocol must also be provided in the manuscript.

Field-specific reporting

Please select the one below that is the best fit for your research. If you are not sure, read the appropriate sections before making your selection.

☒ Life sciences ☐ Behavioural & social sciences ☐ Ecological, evolutionary & environmental sciences

For a reference copy of the document with all sections, see nature.com/documents/nr-reporting-summary-flat.pdf

Life sciences study design

All studies must disclose on these points even when the disclosure is negative.

Sample size	Each group/data point represents 4-16 individual mice (the relevant information is specified under each figure legend).
Data exclusions	No samples were excluded from the analysis.
Replication	All the data were generated from 1-2 independent experiments (number of reproductions are indicated in the relevant figure legends).
Randomization	N/A
Blinding	N/A

Reporting for specific materials, systems and methods

We require information from authors about some types of materials, experimental systems and methods used in many studies. Here, indicate whether each material, system or method listed is relevant to your study. If you are not sure if a list item applies to your research, read the appropriate section before selecting a response.

Materials & experimental systems

n/a	Involved in the study	
	x	Antibodies
x		Eukaryotic cell lines
x		Palaeontology and archaeology
	x	Animals and other organisms
x		Clinical data
x		Dual use research of concern
x		Plants

Methods

n/a	Involved in the study	
x		ChIP-seq
	x	Flow cytometry
x		MRI-based neuroimaging

Antibodies

Antibodies used	B220-FITC (RA3-6B2,Biolegend), CD103-AF700 (2E7,Biolegend), CD11b-PerCp-Cy5.5, BV605 (M1/70, BD Biosciences), CD11c-BV785 (N418, Biolegend), CD19-FITC (1D3/CD19, Biolegend), CD200R1-AF647 (OX-110,BD Biosciences), CD206-PECy7 (C068C2, Biolegend), CD3ε-FITC (17A2, Biolegend), CD4-FITC (RM4-5,Biolegend), CD4-V500 (RM4-5, BD Biosciences), CD64-BV421 (X54-5/7.1,Biolegend), CD8-PerCp-Cy5.5 (53-6.7,Biolegend), CD80-APC/Fire750 (16-10A1,Biolegend), CD86-PE (GL-1, Biolegend), CD90.2-APC Cy7 (53-2.1, BD Biosciences), F4/80-FITC, APC, APC Cy7 (BM8, Biolegend), FoxP3-AF647 (MF23,BD Biosciences), GATA3-PE (TWAJ, eBiosciences), Gr-1-FITC (RB6-8C5, Biolegend), IFN-γ-PE Cy7 (XMG1.2, Biolegend), Ly6C-BV570 (HK1.4, Biolegend), Ly6G-FITC (1A8, BD Biosciences), MHCII-BV510 (M5/114.15.2, Biolegend), Muc5ac (45M1,Invitrogen), NKp46-BV421 (29A1.4, Biolegend),RORγt-BV650 (Q31-378, BD Biosciences), Siglec F-PE (E50-2440, BD Biosciences), T-bet-PE Cy7 (4B10, Biolegend), TCR-β-BV605 (H57-597, Biolegen), TER119-FITC (TER-119, Biolegend), TGF-β1 (polyclonal, Abcam), TNF-α-FITC (MP6-XT22, BD Biosciences) and α-Smooth muscle actin (1A4, Sigma-Aldrich)
Validation	All antibodies were validated according to the suppliers information.

Animals and other research organisms

Policy information about studies involving animals; ARRIVE guidelines recommended for reporting animal research, and Sex and Gender in Research

Laboratory animals	Neonatal BALB/c mice
Wild animals	N/A
Reporting on sex	N/A
Field-collected samples	All mice were housed in SPF conditions at the QIMR Beghofer Medical Research Institute Animal Facility.
Ethics oversight	All experiments were conducted in accordance to the ethics protocols approved by the QIMR Berghofer Medical Research Institute Ethics committee.

Note that full information on the approval of the study protocol must also be provided in the manuscript.

Plants

Seed stocks	N/A
Novel plant genotypes	N/A
Authentication	N/A

Flow Cytometry

Plots

Confirm that:

- [x] The axis labels state the marker and fluorochrome used (e.g. CD4-FITC).
- [x] The axis scales are clearly visible. Include numbers along axes only for bottom left plot of group (a 'group' is an analysis of identical markers).
- [x] All plots are contour plots with outliers or pseudocolor plots.
- [x] A numerical value for number of cells or percentage (with statistics) is provided.

Methodology

Sample preparation	Red blood cells were lysed with Gey's lysis buffer, and the cells re-suspended and washed in FASC buffer (PBS supplemented with 2% fetal calf serum, FCS). Cells were incubated with 2.4G2 antibody for 30 minutes at 40C to prevent non-specific binding, then stained with the surface antibody cocktail for 30 minutes at 40C. The list of antibodies used for flow cytometry is summarized in Table S1. To detect intracellular transcription factors, the cells were first stained with surface antibodies then permeabilized and fixed using FoxP3/transcription factor fixation/permeabilization kit (eBiosciences; CA, USA). After washing with permeabilization buffer, the cells were stained with fluorochrome-conjugated antibodies against ROR-gammaT, GATA3 or Tbet. For intracellular cytokine staining, cells were stimulated with phorbol 12-myristate 13-acetate (PMA; 50 ng/ml) and ionomycin (1 µg/ml) in the presence of brefeldin A (20 µg/ml) at 370C for 3 hours, then washed with FACS buffer and stained with surface antibodies as above. Cells were permeabilized and fixed using FoxP3/transcription factor fixation/permeabilization kit and stained with appropriate antibodies. The samples were acquired on a BD Fortessa IV flow cytometer (BD Biosciences, USA) using the FACSDiva software (version 8, BD Biosciences, USA). Data was analyzed using FlowJo software (Version 10.6; TeeStar, USA) and gating strategy has been depicted in Supplementary Figure S1.
Instrument	BD Fortessa IV flow cytometer (BD Biosciences, USA) and FACSDiva software (version 8, BD Biosciences, USA)
Software	FlowJo software (Version 10.6; TeeStar, USA)
Cell population abundance	Live cells were counted from a a representative aliquot of the single cells suspension.
Gating strategy	*Describe the gating strategy used for all relevant experiments, specifying the preliminary FSC/SSC gates of the starting cell population, indicating where boundaries between "positive" and "negative" staining cell populations are defined.*

- [x] Tick this box to confirm that a figure exemplifying the gating strategy is provided in the Supplementary Information.
